# The Problem of Metal Needles in Acupuncture-fMRI Studies

**DOI:** 10.1155/2011/808203

**Published:** 2011-03-01

**Authors:** Florian Beissner, Ulrike Nöth, Thomas Schockert

**Affiliations:** ^1^Brain Imaging Center, Goethe University, 60325 Frankfurt, Germany; ^2^Department of Neurology, Goethe University, 60325 Frankfurt, Germany; ^3^Department of Chinese Medicine, Witten/Herdecke Private University, 58448 Witten, Germany

## Abstract

Acupuncture is a therapy based on sensory stimulation of the human body by means of metal needles. The exact underlying mechanisms of acupuncture have not been clarified so far. Functional magnetic resonance imaging (fMRI) has become an important tool in acupuncture research. Standard acupuncture needles, which are made of ferromagnetic steel, however, are problematic in acupuncture-fMRI studies for several reasons, such as attraction by the scanner's magnetic field, significant image distortions and signal-dropouts, when positioned close to the head or even heating due to absorption of radio frequency (RF). The aim of this study was to compare two novel types of acupuncture needles with a standard needle for their effect on MRI image quality. The standard needle severely reduced image quality, when located inside the RF coil. The nonferromagnetic metal needle may pose a risk due to RF heating, while the plastic needle has a significantly larger diameter. In conclusion, our recommendations are: (1) standard needles should not be used in MRI; (2) Nonferromagnetic metal needles seem to be the best choice for acupoints outside of the transmitter coil; and (3) only plastic needles are suited for points inside the coil. Laser acupuncture may be a safe alternative, too.

## 1. Introduction

Functional magnetic resonance imaging (fMRI) has been used since the late nineties to study possible underlying mechanisms of acupuncture [1, 2]. In the meantime, more than 70 studies have been conducted, producing a large variety of results [3]. In a typical experimental setting, acupuncture stimulation is applied while the subject is being scanned, which necessitates the use of acupuncture needles inside or close to the magnet of the MR scanner. As standard acupuncture needles are usually made of ferromagnetic steel, this poses an important problem for several reasons. (1) Like all ferromagnetic objects, these needles experience a force in the magnetic field of the MR scanner, which is dependent on the magnetic field strength and directed towards the area of highest homogeneity of the field. As this area lies inside the scanner bore, where the head of the subject is located, a loose needle poses a threat especially for the eyes of the subject. (2) Ferromagnetic objects severely distort the scanner's static magnetic field. While this is a minor problem when the needle stays outside of the bore, it can lead to significant image distortions and signal drop-outs if the needle is close to the investigated area. (3) As acupuncture needles are also conductors for electric currents, they can in theory heat up during radio frequency (RF) transmission inside the RF transmit coil. This risk of heating increases further, if leads are attached as in electro-acupuncture. 

For these reasons, most scientists working in the field have so far restricted their choice of stimulation sites to the body's extremities. However, a number of important acupuncture points are situated at the torso or even on the head. In addition to classical points from Chinese body acupuncture, a number of so-called microsystems have to be mentioned here: Chinese ear acupuncture [4, 5], French ear acupuncture [6], Chinese scalp acupuncture [7], and Yamamoto new scalp acupuncture (YNSA) [8, 9]. So far, acupuncture points of these systems have hardly been used in acupuncture-fMRI studies [10], although they may be interesting targets because of the direct stimulation of cranial nerves.

Another possibility to avoid MR-related problems is to use needles made from non-ferromagnetic materials like silver or gold. However, most available needles are only silver- or goldplated and still have a steel core. On the other hand, pure silver or gold needles are very soft and tend to bend upon insertion, making their application in an MR experiment, where timing is extremely important, problematic. Furthermore, pure silver or gold one-way needles are expensive and can only be obtained from a few manufacturers in Japan (e.g., Asashi Industry, Kawaguchi; Maeda Toyokichi Shoten, Tokyo). It should be mentioned that silver and gold needles are often very soft which makes reliable machining without burrs on the metal surface impossible. Upon insertion, these burrs can cause a sharp pain in subjects. A further option is to use titanium needles, which are stiffer than sliver and gold needles. They can be used with thinner gauges and machined with a smoother surface. Unfortunately, titanium needles are hardly on the market as no manufacturer is producing them for general sale at the moment.

The aim of this study was (1) to compare two novel types of experimental acupuncture needles (non-ferromagnetic metal and plastic) with a standard ferromagnetic needle for their effect on image quality in anatomical and functional MRI, (2) to give some advice on safety issues in relation to the use of electrically conducting needles, and (3) to name the advantages and disadvantages of the different needle types which might help to choose the most appropriate needle for a specific experimental set-up.

## 2. Materials and Methods

The study was conducted following the Declaration of Helsinki on Biomedical Research Involving Human Subjects. The University of Frankfurt Medical Faculty Ethics Review Board approved the study protocol. Informed written consent was obtained from the subject.

### 2.1. Needle Types

**Figure 1:**
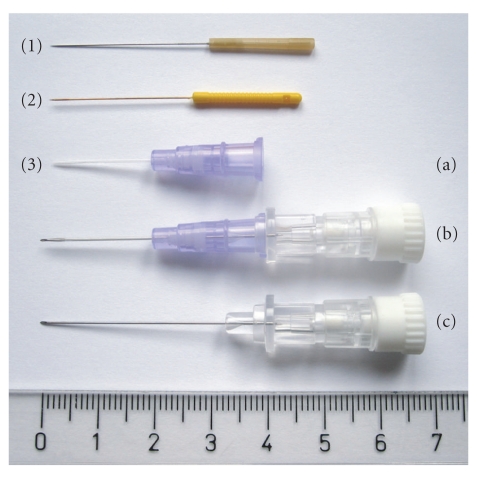
The different acupuncture needles compared in this study: (1) standard acupuncture needle, (2) non-ferromagnetic needle, and (3) modified peripheral I.V. catheter: (a) catheter without mandrin, (b) catheter with mandrin, (c) steel mandrin (to be removed after insertion and before application in MR environment).

In this study, we tested three types of needles for their applicability in the MR environment and their effect on MR image quality: a standard acupuncture needle made from ferromagnetic steel, a custom-made non-ferromagnetic metal needle, and a modified peripheral I.V. catheter (original idea by Schockert et al. [11]). For details see Table 1 and Figure 1.

### 2.2. MRI Measurements

All measurements were performed on a whole body 3T Magnetom TRIO Scanner (Siemens, Erlangen, Germany) equipped with a body coil for RF transmission and an 8-channel phased array receive only head coil. For fMRI, a T2*-weighted gradient-echo Echo Planar Imaging (EPI) sequence [12] with a TR of 2500ms, a TE of 30 ms, a FOV of 192 mm × 192 mm, and a flip angle of 90 degrees was used. The matrix size was 64 × 64, resulting in an in-plane resolution of 3 × 3 mm^2^. A total of 38 axial slices (thickness 2.5 mm, gap 1.25 mm) were collected for whole brain coverage. Ten consecutive volumes were acquired and a mean image was calculated using FSL 4.1 (FMRIB, Oxford). For anatomical reference, a T1-weighted MP-RAGE sequence [13] with an isotropic resolution of 1mm was collected. 

Prior to the measurement, the needle was placed on a cotton swab and attached securely to the head with adhesive tape directly above the YNSA point “basal ganglia”, which is located on the midline shortly above the hairline [8]. The needle was not allowed to touch the skin in order to prevent injury by possible heating up of the needle. 

## 3. Results

**Figure 2:**
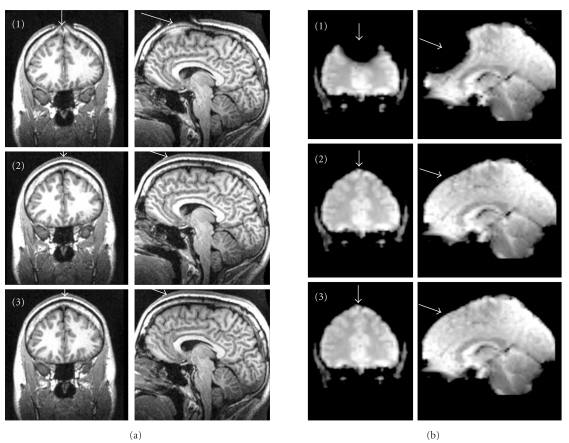
(a) Structural images (MP-RAGE). (b) Functional images (EPI). Top row: standard needle (1) causing severe image distortions as well as areas of complete signal loss; middle row: non-ferromagnetic needle (2); bottom row: modified peripheral I.V. catheter (3). Both non-ferromagnetic alternatives (2, 3) did not disturb the image acquisition.

Figure 2 shows the image quality results for the three needle types. The standard ferromagnetic acupuncture needle caused severe distortions in the anatomical image (MP-RAGE) as well as distortions and signal drop-outs in the EPI image. In contrast, none of the other two needles caused visible distortions or drop-outs in any of the measurements. We encountered no further problems when using the needles inside of the head coil.

Judging from the image quality results, it is evident that both the non-ferromagnetic needle and the modified catheter can be used to administer acupuncture stimulation in the MR scanner at sites close to or even on the head. 

## 4. Discussion

Although we did not encounter any problems other than the degraded image quality in this study, some safety considerations are required with respect to the metal needles. In principle, all electrically conducting material can act as antenna and consequently heat up due to eddy currents induced by fluctuating electromagnetic fields such as the RF used for spin excitation or the magnetic field gradients used for spatial encoding. As shown in a previous study, the material surrounding the wire tip is particularly prone to RF heating, especially for decreasing wire diameter [14]. This effect can lead to temperature increases of more than 60°C when resonance conditions for the antenna effect are met [15] with the strongest heating at the tips. This is the case when the length of the conductor inside the RF coil corresponds to a multiple of the resonant length, which is 1.17 m at 3 Tesla. Although heating has been shown to be noticeable for a conductor length of somewhat less than half the resonant length, there are hardly any effects for shorter conductors as shown by Dempsey et al. [15]; that is, the probability of significant heating of a needle of approximately 5 cm is very low. However, the danger of heating resulting in injury increases considerably when conducting cables are attached for electrostimulation via the acupuncture needle. Firstly, the total length of the conductor increases by the length of the cable which could come closer to a resonant length where heating might occur. Secondly, the effect of electromagnetic induction heating can occur when a circuit in (or close to) resonance is formed due to loop formation, with temperature increases of more than 60°C [15]. Thus, if electro-stimulation is to be carried out in an MR environment, great care has to be taken to ensure subject safety. An analogous problem is encountered in simultaneous EEG-MRI acquisitions, so the same safety considerations are valid and the safety devices proposed for EEG-MRI [16, 17] should be adapted appropriately. However, a detailed discussion of the technical requirements would be beyond the scope of this paper. 

In contrast, magnetic field gradients that are used for spatial encoding do not pose a heating problem due to the following reasons: (1) the switching frequency of the gradients is in the kHz-range, that is, a factor of 1000 lower than the RF frequency (which is in the MHz range for clinical MR scanners), so the resonant length exceeds the needle size by a factor of 10^3^ to 10^4^; (2) typical gradient switching rates are in general low to avoid peripheral nerve stimulation.

In summary, the following advantages and disadvantages for the three needle types were found: the standard ferromagnetic acupuncture needle is the cheapest alternative. However, it cannot be recommended for acupuncture-fMRI studies, unless the stimulation site lies at the very distal part of the extremities. Even so, a loose needle can become a threat for the subject if accelerated towards the subject's head. The non-ferromagnetic needle can be used exactly like a standard needle. It is suitable for acupuncture-fMRI studies, even for ear and scalp acupuncture. Due to its short length, the risk of RF heating is low, but cannot be wholly excluded. So far, no acupuncture-fMRI study has reported burns due to heating of needles inside the MR scanner. However, if conducting cables are attached for electro-stimulation, the risk of heating, resulting in injuries is considerably increased, as described above. Due to these facts and as several other experimental parameters (the discussion of which is beyond the scope of this paper) influence heating, extensive safety testing is highly recommended before performing acupuncture experiments in an MR scanner. In particular, only sequences with a low specific absorption rate (SAR), that is, low levels of RF power should be used, such as gradient-echo EPI for fMRI and T1-weighted gradient-echo sequences with low excitation angles for anatomical reference.

The modified catheter is nonmagnetic and nonconducting. Therefore, it is perfectly suited for acupuncture-fMRI studies. However, due to its relatively large diameter of 0.64 mm, its application can be considerably more painful. It should therefore not be used for acupuncture of sensitive areas such as the ear. 

**Figure 3:**
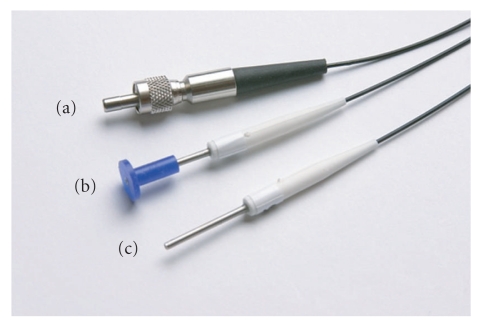
Laser acupuncture is another safe alternative to the use of needles in the MR environment. This image shows the Laserneedle device, where the laser beam is guided to the acupuncture point by means of fibre optical cables. (a) Connector between cable and laser (outside of the scanner room); (b) optode with silicone adapter for application on the skin; (c) optode without adapter. (This image shows a non-MR-safe version of the laser.)

It should be noted that apart from the choice of appropriate needles, further problems need to be solved when using acupoints located on the head: a major issue is the difficulty of manipulating the needles inside the scanner. A possible solution might be laser acupuncture [18]. In our research facility, we have frequently used a custom-made laser acupuncture system (Laserneedle EG, Wehrden, Germany), which consists of a multichannel diode laser (685 nm, 40 mW) with fiber optical cables and optodes attached to the skin with a silicone adapter and adhesive tape (see Figure 3). In contrast to hand held laser acupuncture devices, this system allows a simultaneous stimulation of any acupuncture point combination and even fulfils the requirements to perform double-blind investigations. For use in the MRI, the optodes were modified to prevent interactions with the magnetic field. The fibre optical cables of the laser needle optodes were fed through a waveguide in the Faraday cage of the scanner so the system could be operated from the outside. Other groups have successfully used laser acupuncture in fMRI studies, too [19–21]. However, it should be noted that although several studies suggest a similar effect of laser and conventional acupuncture [21–26], their equivalence has not yet been fully established.

In summary, among the three needle types tested, the non-ferromagnetic model seems to be the best choice for the application in acupuncture-fMRI studies, if stimulation points are situated on the torso or neck but outside of the RF transmitter coil. For points inside the RF coil, we recommend to use the modified catheter approach. Laser acupuncture may be a safe alternative, too.

In conclusion, we hope that the approaches described in this study will lead to new fMRI studies of acupuncture points on the torso as well as on the microsystems on the head.

## Figures and Tables

**Table 1:** Technical specifications of the different acupuncture needles compared in this study.

	Type	Manufacturer	Material	Diameter
(1) Standard acupuncture needle	B-Type	Seirin Cooperation, Shizuoka, Japan	Stainless steel, plastic handle	0.30 mm
(2) Non-ferromagnetic needle	PL-XC 3025 (for clinical tests only)	Moxom Acupuncture GmbH, Graefenhainichen, Germany	Copper alloy, plastic handle	0.30 mm
(3) Modified peripheral I.V. catheter	26G, Versatus-W, SR+DU2619WX	Terumo Europe N.V., Leuven, Belgium	Polyurethane	0.64 mm

## References

[1] Cho ZH, Chung SC, Jones JP (1998). New findings of the correlation between acupoints and corresponding brain cortices using functional MRI. *Proceedings of the National Academy of Sciences of the United States of America*.

[2] Hui KKS, Liu J, Kwong KK (1997). Functional mapping of the human brain during acupuncture with magnetic resonance imaging somatosensory cortex activation. *World Journal of Acupuncture-Moxibustion*.

[3] Beissner F (2010). fMRI studies of acupuncture mechanisms—a critique. *Fact*.

[4] Cheng X, Deng L, Gan Y, He S, Ji X, Li Y (1999). *Chinese Acupuncture and Moxibustion*.

[5] Gori L, Firenzuoli F (2007). Ear acupuncture in European traditional medicine. *Evidence-Based Complementary and Alternative Medicine*.

[6] Nogier R (2009). *Auriculotherapy*.

[7] Shao S, Wei H (2006). *Chinese-English Illustrated Meridians and Acupoints*.

[8] Yamamoto T, Yamamoto H (1998). *Yamamoto New Scalp Acupuncture*.

[9] Yamamoto T, Yamamoto H (2010). *Yamamoto New Scalp Acupuncture YNSA*.

[10] Schockert T, Schnitker R, Boroojerdi B (2010). Cortical activation by Yamamoto new scalp acupuncture in the treatment of patients with a stroke: a sham-controlled study using functional MRI. *Acupuncture in Medicine*.

[11] Schockert T, Schnitker R, Kastrau F New acupuncture needle for magnetic resonance research.

[12] Mansfield P (1977). Multi-planar image formation using NMR spin echoes. *Journal of Physics C*.

[13] Mugler JP, Brookeman JR (1990). Three-dimensional magnetization-prepared rapid gradient-echo imaging (3D MP RAGE). *Magnetic Resonance in Medicine*.

[14] Armenean C, Perrin E, Armenean M, Beuf O, Pilleul F, Saint-Jalmes H (2004). RF-induced temperature elevation along metallic wires in clinical magnetic resonance imaging: influence of diameter and length. *Magnetic Resonance in Medicine*.

[15] Dempsey MF, Condon B, Hadley DM (2001). Investigation of the factors responsible for burns during MRI. *Journal of Magnetic Resonance Imaging*.

[16] Lemieux L, Allen PJ, Franconi F, Symms MR, Fish DR (1997). Recording of EEG during fMRI experiments: patient safety. *Magnetic Resonance in Medicine*.

[17] Carmichael DW, Thornton JS, Rodionov R (2010). Feasibility of simultaneous intracranial EEG-fMRI in humans: a safety study. *NeuroImage*.

[18] Whittaker P (2004). Laser acupuncture: past, present, and future. *Lasers in Medical Science*.

[19] Siedentopf CM, Koppelstaetter F, Haala IA (2005). Laser acupuncture induced specific cerebral cortical and subcortical activations in humans. *Lasers in Medical Science*.

[20] Siedentopf CM, Golaszewski SM, Mottaghy FM, Ruff CC, Felber S, Schlager A (2002). Functional magnetic resonance imaging detects activation of the visual association cortex during laser acupuncture of the foot in humans. *Neuroscience Letters*.

[21] Litscher G, Rachbauer D, Ropele S (2004). Acupuncture using laser needles modulates brain function: first evidence from functional transcranial Doppler sonography and functional magnetic resonance imaging. *Lasers in Medical Science*.

[22] Gottschling S, Meyer S, Gribova I (2008). Laser acupuncture in children with headache: a double-blind, randomized, bicenter, placebo-controlled trial. *Pain*.

[23] Litscher G, Schikora D (2002). Cerebral vascular effects of non-invasive laserneedles measured by transorbital and transtemporal Doppler sonography. *Lasers in Medical Science*.

[24] Banzer W, Hübscher M, Seib M, Vogt L (2006). Short-time effects of laser needle stimulation on the peripheral microcirculation assessed by laser Doppler spectroscopy and near-infrared spectroscopy. *Photomedicine and Laser Surgery*.

[25] Butkovic D, Toljan S, Matolic M, Kralik S, Radešić L (2005). Comparison of laser acupuncture and metoclopramide in PONV prevention in children. *Paediatric Anaesthesia*.

[26] Litscher G, Xie Z, Wang L, Gaischek I (2009). Blue 405 nm laser light mediates heart rate—investigations at the acupoint Neiguan (Pe. 6) in Chinese adults. *North American Journal of Medical Sciences*.

